# Investigating Localized Electrochemical of Ferrocenyl-Imidazolium in Ionic Liquid Using Scanning Electrochemical Microscopy Configuration

**DOI:** 10.3390/molecules27186004

**Published:** 2022-09-15

**Authors:** Thuan-Nguyen Pham-Truong, Jalal Ghilane

**Affiliations:** 1 Université Paris Cité, CNRS, ITODYS, F-75013 Paris, France; 2 Cy Cergy Paris University, LPPI 5 Mail Gay Lussac, F-95000 Cergy, France

**Keywords:** electrochemistry, room temperature ionic liquid, ultramicroelectrode, scanning electrochemical microscopy (SECM), thin layer

## Abstract

In the present work, the localized electrochemical behavior of redox molecule in ionic liquid has been investigated using scanning electrochemical microscopy. The electrochemical response of ferrocenyl-imidazolium redox mediator was studied by recording approach curves over a conducting and insulating substrate in an undiluted ionic liquid. The SECM approach curve over the conducting substrate displays a positive feedback, as observed in classical solvent. However, in the case of the insulating substrate, the approach curve reveals different shapes, depending on the used approach speed. In this configuration, low approach speed is necessary to reach the expected negative feedback. Interestingly, at a very close distance between the UME and the insulating substrate, a thin film behavior is revealed. In addition, the approach curves on both insulator and conducting substrates can be reconstructed from punctual responses at different distance tip-substrate. The latter match perfectly with the expected theoretical curves over conducting and insulating under diffusion control.

## 1. Introduction

Since the beginning of the last decade, ionic liquid has been widely investigated as green solvent for a large spectrum of applications, such as Li-ion batteries [1,2], dye-sensitized solar cell [3,4], electrodeposition [5], catalysis [6], etc. Resulting from its unique structure, IL possesses a negligible vapor pressure, a large liquidus range, and a high thermal and chemical resistance [7,8]. From the electrochemical point of view, ionic liquid acts as a solvent and electrolyte with a large accessible electrochemical window, comprising the range of 3.5 V to 6 V [9]. Numerous, up-to-date works were reported, mentioning the mass transport and the heterogeneous electron transfer of various redox-active species in ionic liquid [10,11,12,13,14]. Concerning the mass transfer of redox species inside ionic liquid solution, different approaches were performed by providing comparable results, typically 3.9 × 10^−7^ cm^2^.s^−1^ for ferrocene in ionic liquid ([EMI^+^][TF_2_N^−^]) [15]. This value is much lower than the value found in acetonitrile solution (D = 2 × 10^−5^ cm^2^.s^−1^) that causes non-negligible change of the electrochemical response of the redox species. Similarly, the use of ionic liquid in electrochemical measurement reveals some unexpected behaviors, such as the inequality of the diffusion coefficient. This has been evidenced using ultramicroelectrode and scanning electrochemical microscopy (SECM) and was reported for oxygen and nitrophenyl derivatives [16,17,18]. Recently, SECM was employed to investigate the localized electrochemical response of redox mediator in ionic liquid. Overall, the SECM approach curve presents similar approach curves over a conducting substrate, however, a large change in the approach recorded on the insulating substrate was reported. The latter was attributed to the viscosity of the ionic liquid through the contribution of the convection into the mass transport [19]. Nkuku and LeSuer [20] proposed a new SECM model in deep eutectic solvent via the introduction of the convection term on the total mass transfer during the tip’s approach. Several examples have been reported with strong SCEM applications, including the investigation of transport processes through coating and membranes, heterogeneous catalysis, energy storage and conversion, and biological applications [21,22].

In this context, the deep understanding of the electron transfer and the mass transport at the surface of a material provides a considerable role for improving the efficiency of different electrochemical processes involved in ionic liquid media. The present work reports the investigation of the SECM responses using redox molecule-based ionic liquid in undiluted ionic liquid solution. Different approaches curves were recorded on conducting and insulating substrates. In addition, the approach curves could be reconstructed from the steady state electrochemical response at the UME, recorded at different UME-substrate distances. Finally, the electrochemical SECM response over an insulating substrate reveals thin film electrochemical cell behaviors.

## 2. Results and Discussions

**Figure 1:**
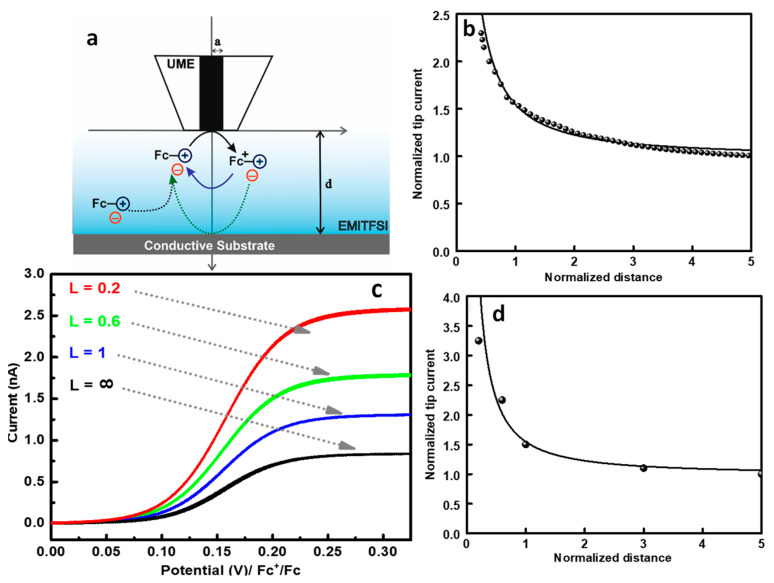
(**a**) Scheme illustrating the SECM feedback over a conducting substrate. (**b**) Approach curve recorded on Pt UME in 10 mM of Fc-IL in [EMIm^+^][TF_2_N^−^] solution (dot) experimental curve, (solid line) theoretical curve. (**c**) Cyclic voltammograms at different distance tip-substrate (L = d/a): (black) L = ∞, (blue) L = 1, (green) L = 0.6, (red) L = 0.2. (**d**) Reconstructed approach curve from the steady-state CV at a potential 0.3 V vs. Fc^+^/Fc.

**Figure 2:**
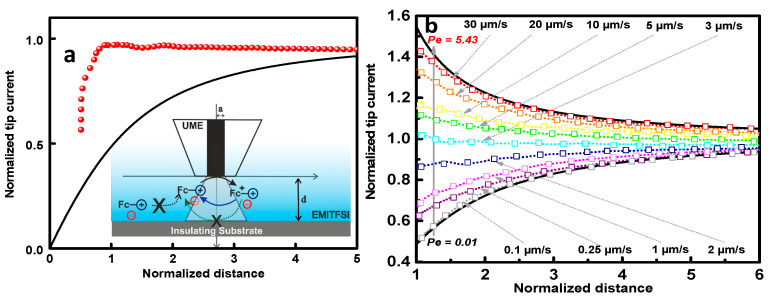
(**a**) Approach curve recorded on Pt UME over insulating substrate (glass) in 10 mM of Fc-IL in [EMIm^+^][TF_2_N^−^] solution (red dot, experimental curve and solid line theoretical curve). (**b**) Experimental approach curves (square) recorded with different approach speed ranging from 30 to 0.1 µm.s^−1^, (solid line theoretical curves for conducting and insulating substrate).

An ultramicroelectrode (UME), attached to a vertical axis piezo of a SECM setup, was approached to the substrate through an ionic liquid solution ([EMI^+^][TF_2_N^−^]) containing 10 mM of 1-ferrocenylmethyl-3-methyl imidazolium Tf_2_N, Fc-IL, as redox mediator (Figure 1a). The approach curve recorded on conducting substrate displays a current enhancement when the tip approaches the substrate (Figure 2b). This shape is related to the generation of the oxidized Fc-imidazolium at the conducting substrate, and thus generating a positive feedback. In addition, the experimental approach curve matches the theoretical one expected for conducting substrate under diffusion control. This result demonstrates that despite its viscosity, the approach curve recorded in the presence of Fc-IL in ionic liquid over a conducting substrate is similar to that observed in classical solvents, e.g., acetonitrile.

Furthermore, the electrochemical signals provided by the redox mediator were recorded at different distances between the tip and the substrate (L). Indeed, independent to the distance, L, the recorded cyclic voltammograms display a steady-state current, as shown in Figure 1c. From the value of the steady-state current in the bulk solution, the diffusion coefficient of the [FcMIm^+^M][TF_2_N^−^] can be calculated via the following equation [23].

(1) i=4nFDCa

where i represents the steady-state current, n the number of exchanged electrons, F the Faraday constant (96,500 C·mol^−1^), D (cm^2^.s^−1^) the diffusion coefficient of the mediator, *C* (mol.cm^−3^) the bulk concentration of the mediator and a (cm) the radius of the UME. As shown in the Figure 1c, the CV at L = ∞ represents a well-defined diffusion-controlled system with a plateau current. The measured diffusion coefficient in [EMI^+^][TF_2_N^−^] solution is D_[FcMIm_^+^_M] [TF2N_^−^_]_ = 4.6 × 10^−7^ cm^2^.s^−1^, which is two orders of magnitude lower than the one measured in acetonitrile solution. This difference is attributed to the higher viscosity of the ionic liquid. For the other tip-substrate distances, the steady state shape is maintained, but the plateau current increases when the tip is getting close to the conducting substrate. The current increase is related to the generation of the Fc-IL at the substrate. From different voltammograms recorded at different tip-substrate distances, the approach curve could also be reconstructed by plotting the value of the steady-state current (at E = 0.3 V vs. Fc^+^/Fc) as a function of the tip-substrate distance (Figure 1d). The latter matches perfectly the theoretical approach curve expected for a tip approaching conducting substrate under diffusion control. Overall, the approach curve over a conducting substrate in ionic liquid is not affected by the viscosity of the ionic liquid and the lower diffusion coefficient.

Similar experiments as reported above were performed over the insulating substrate and the obtained results are illustrated in Figure 2a.

The approach curve displays a current decrease when the tip is brought closer to the insulating substrate, which is attributed to the absence of backward electrochemical reaction at the substrate, and thus the absence of the regeneration of the Fc-IL redox mediator. However, compared to the theoretical approach curve expected for the insulating substrate, a large deviation is observed, as shown in Figure 2b. This change is probably due to the high viscosity of the ionic liquid (35.55 cP at 25 °C [24]).

In highly viscous solvents, as in this case, ionic liquid EMITFSI, the phenomenon of diffusion is strongly affected. Indeed, to get insight the mass transfer in such a solvent, approach curves were performed at different approach speeds, as shown in the Figure 2b. The combination of the diffusion and the convection is ensured by Peclet number, which is equal to νa/D, where ν is the tip velocity, a represents the tip radius and D is the diffusion coefficient. For tip velocity ranging from 0.3 µm.s^−1^ to 30 µm.s^−1^ (Pe = 0.1–5.43), the approach curves onto insulating substrate deviate from fully negative to positive-like feedback, where the contribution of the convection becomes dominated in the mass transport. However, at an approach rate around 0.1 µm.s^−1^ (Pe = 0.01), the feedback curve fits the theoretical approach curve expected for the insulating substrate. This experiment highlights the influence of the viscosity of the ionic liquid when recording the approach curve onto the insulating substrate. Unlike classical solvents, the SECM approach curve in ionic liquid over the insulating substrate should be performed at a lower approach rate, typically around 0.1 µm.s^−1^.

**Figure 3:**
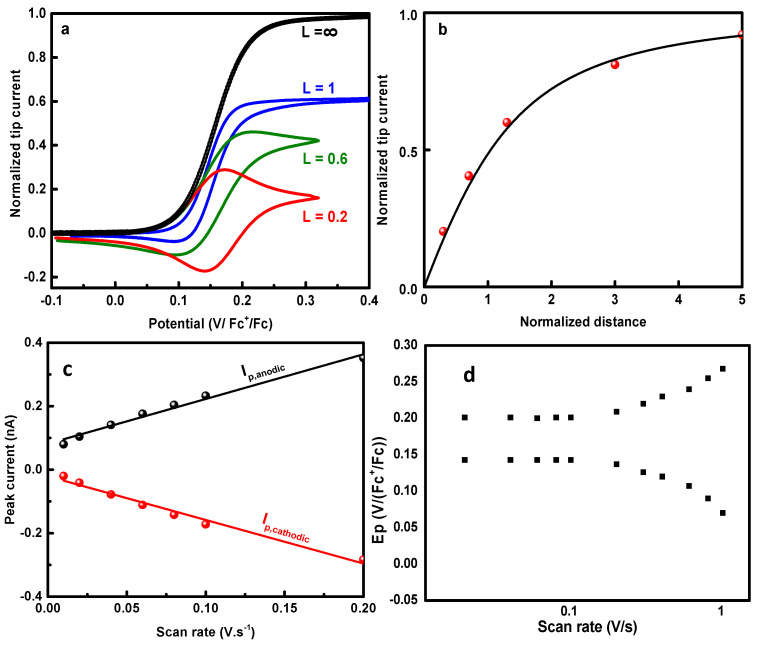
(**a**) Cyclic voltammograms at different distance tip-substrate (L = d/a): (black) L = ∞, (blue) L = 1, (green) L = 0.6, (red) L = 0.2. (**b**) Reconstruction of the approach curve by means of the plateau currents collected from chronoamperometry at different tip-insulating substrate distances (red dots), solid lines correspond to the theoretical approach curve. (**c**) Variation of the anodic and cathodic peaks current as function of the scan rate. (**d**) Variation of the anodic and cathodic peaks potential in function of the scan rate (Log plot).

To get further insight, the CVs at different tip-substrate distances were recorded, as shown in Figure 3a. Far from the substrate, typically tip-substrate distance higher than 3a, steady-state CV is obtained, indicating that the mass transport is controlled by the radial diffusion.

At L = 1, the steady-state current is still observed and the current plateau is lower than that obtained far from the substrate. The current plateau decreases are due to the insulating substrate, which lowers the diffusion of the Fc-IL mediator to the UME. For normalized distance L = 0.6, the shape of the recorded CV deviates from classical steady-state and an oxidation peak start to be visible at potential around 0.2 V vs. Fc^+^/Fc followed by a plateau current. During the backward scan, a small peak is observed. This behavior suggests changes in the diffusion regime at the UME. For closer tip-substrate distance L = 0.2, the CV displays unexpected form with a well-defined reversible peak shape. At this position, oxidation and the reverse reduction peaks of Fc-IL are observed with a peak-to-peak separation less than 20 mV with a current ratio close to 1. These electrochemical signatures are similar to characteristics of thin film configuration. The phenomenon that can be occurred in this configuration is mainly due to the high viscosity of the ionic liquid. The proposed behavior can induce important effects to the electrochemical response at the UME, where the diffusion of the redox mediator from and to the electrode surface is inhibited by amplifying the physical obstruction due to the presence of the UME. Perhaps, a meta-state is generated in which all the Fc-IL molecules are confined in the small volume defined by the UME and the substrate. This situation is similar to that observed for thin-layer electrochemical cell, where a redox probe is trapped between two parallel electrodes separated by a small gap [25]. Therefore, at the reverse scan, the generated oxidized species are localized in the vicinity of the electrode and susceptible for reduction without any overpotential (ΔE < 20 mV). In addition, a linear relationship between the anodic and cathodic peak currents, as a function of the scan rate is obtained confirming the presence of non-diffusive regime with a quasi-immobilized electroactive layer (Figure 3c). Furthermore, the electron transfer rate constants at the interface was evaluated using Laviron’s formalism [26].

k_app_ = (1 − α)nFυ_a_/RT

where α is the charge-transfer coefficient and υ_a_ is the intersect of two linear regions in the plot Ep vs. log(v) (Figure 3d). The calculated electron transfer rate constant displays a value of 26 s^−1^, which is similar to that reported for attached Fc head-group onto electrode surface [27,28,29].

The cyclic voltammograms at different scan rates offer the real behaviors of the redox species in the vicinity of the microelectrode. Finally, a more reliable approach curve by eliminating the contribution of the convection is reconstructed using the steady-state current observed during the chronoamperometry, at 0.4 V vs. Fc^+^/Fc during 50 s, at different tip-substrate distance. Interestingly, the variation of the normalized current i_T_/i_∞_, as a function of the normalized distance tip-substrate, is plotted as shown in the Figure 3b. The evolution of the normalized current matches perfectly with the theoretical approach curves, which are calculated under steady-state conditions. This result shows the possibility to record approach curves in ionic liquid under diffusion control.

## 3. Materials and Methods

Chemicals. All chemical products were purchased from Sigma-Aldrich and used without further purifications. The 1-ferrocenylmethyl-3-methyl imidazolium bis(trifluoromethane)sulfonimide (Fc-IL) was synthetized according to previously reported work [30]. Briefly, 1-ferrocenylmethyl-3-methyl imidazolium iodide was synthetized by refluxing dichloromethane solution containing 1-ferrocenylmethyl imidazole (1.68 g, 10 mmol) in presence of methyl iodide (1.9 mL, 30 mmol) for 2 h. The solvent and non-reacted compound was evaporated under reduced pressure affording brown viscous oil (Yield = 99%). Then, 1-ferrocenylmethyl-3-methyl imidazolium Tf_2_N was achieved via ion exchange at 70 °C in aqueous solution containing as-prepared molecule and an excess of Lithium bis(trifluoromethanesulfonyl)imide (LiTf_2_N) for 24 h. The organic phase was collected and dried over MgSO_4_.

Electrochemical measurements. Scanning electrochemical microscopy measurements were performed with a special-purpose cell in which a silver wire and a platinum wire were used as reference and auxiliary electrode, respectively. Commercial platinum microelectrode with a diameter of 10 µm was used as a working electrode, Pt rod electrode (1 mm) and glass slide were used as the substrates. The electrochemical measurements were performed using a CHI920C bipotentiostat (CH Instruments, Austin, TX, USA).

## 4. Conclusions

In summary, the SECM investigations of Fc-IL in ionic liquid over conducting and insulating substrates have been performed. The results confirm that the approach curve recorded over conducting substrate displays a positive feedback. The approach curve is not affected by the high viscosity of the ionic liquid and the low diffusion coefficient of Fc-IL, confirming the fast regeneration of the mediator over the conducting substrate. However, for insulating substrate, the approach curve deviates from the expected negative feedback. This effect is related to the high viscosity of the ionic liquid, and thus the contribution of mass transport and convection. The latter could be suppressed by performing the approach curve at lower speed (typically in the order of 0.1 µm.s^−1^). Interestingly, the expected steady-state CV, recorded at the UME close to the insulating substrate (L = 0.2), displays a peak shape CV, suggesting the formation of extremely thin layer of Fc-IL in EMITFSI solution. The low UME-substrate distance and the high viscosity of the ionic liquid confine the Fc-IL molecules in the small volume defined by the UME and the substrate. Finally, the approach curve over insulating the substrate was reconstructed, leading to a totally negative feedback and suppressing the convection part. This work paves the way for understanding the redox phenomena at the interface between ionic liquid solution and the electrode surface for further applications in energy storage and conversion.

## Data Availability

Not applicable.

## Sample Availability

Samples of the compounds are available from the authors.
